# Need of education for dry powder inhaler storage and retention – a patient-reported survey

**DOI:** 10.1186/s40248-016-0057-0

**Published:** 2016-06-08

**Authors:** Birger Norderud Lærum, Gunilla Telg, Georgios Stratelis

**Affiliations:** Department of Clinical Science, University of Bergen, N-5020 Bergen, Norway; Department of Thoracic Medicine, Haukeland University Hospital, Bergen, Norway; AstraZeneca NordicBaltic, Södertälje, Sweden

**Keywords:** Real life, Survey, Dry powder inhaler, Asthma, Control, Storage, Retention, Compliance

## Abstract

**Background:**

Dry powder inhalers (DPIs) are the most commonly used devices in asthma treatment in the Nordic countries. As new DPIs become available, patients are likely to be exposed to more than one type of device, with variable optimal handling. The aim was to examine real life storage and retention of multidose DPIs in patients with asthma.

**Methods:**

This patient-reported survey on real life storage and retention of DPIs included asthma patients using multidose DPIs. Basic patient characteristics, information on inhaler use and storage, check of expiry date, and concurrent inhaler use was examined using an on line questionnaire.

**Results:**

A total of 738 patients were included with a median age of 41 years, out of which 83 % were women. Sixty-three per cent reported storage conditions pre-defined as risk locations for their maintenance inhaler and 38 % of the responding patients had more than one maintenance inhaler in use at the same time. Two thirds of the study population checked inhaler expiry date less than monthly or not at all. Use after expiry date was frequently reported. Two thirds of the patients had not received information on DPI storage, either from their doctor and/or nurse or at the pharmacy.

**Conclusions:**

This patient reported survey indicates that two thirds of the patients store their inhaler devices in suboptimal conditions, and only a minority had received instruction regarding inhaler handling. Non awareness of inhalers’ expiry dates and use of more than one maintenance inhaler simultaneously was common. As inhaler mishandling may impact device functionality, improved communication and patient education is needed.

## Background

A number of different devices are used for drug delivery to the lungs in the treatment of asthma, e.g. dry powder inhalers (DPIs), pressurized metered dose inhalers (pMDIs) and soft mist inhalers (SMIs). Of these, DPIs are the most commonly used inhalers in the Nordic countries. In Norway, the past years have witnessed an increase in available DPI devices to currently 12 different device providers. Combinations of inhaled corticosteroids (ICS) and long-acting beta_2_ agonists (LABA) were the top two reimbursed prescribed drugs in 2012 [1]. Hence, treatment of asthma have a substantial impact on health care costs.

The asthma prevalence has increased in Norway over the last four decades, and is reported to be between 8 and 12 % [2, 3]. GINA guidelines recommend a stepwise approach to reach optimal asthma control [4]. This should include not only receiving a correct diagnosis, but also a choice of inhaled therapy made in agreement between the physician and the patient, training on correct device use [5], and information on inhaler handling, storage, and retention. On the other hand, lack of treatment adherence and incorrect daily use may occur in up to 94 % of asthma patients [6, 7]. Hence, each of these steps are important to achieve asthma control.

Due to effective modern inhalation treatment, patients can today live a normal active life, and may have multiple inhalers available for use in different locations according to their lifestyle. Asthma patients are likely to be exposed to more than one type of inhaler concurrently during their treatment journey. Suboptimal inhaler technique [8] and inhaler mishandling [9] are still common in real life use. Lack of device continuity may increase these problems [10, 11]. To ensure optimal inhaler use, patient's preference [12] and education [13] and training of health care professionals in device handling [5] need to be in focus. The fact that inhalers differ in inhalation techniques, time in-use, and storage conditions needs increased awareness.

DPIs are breath actuated and more easy to use as there is no need to coordinate the inhalation, in contrast to most pMDIs. A potential drawback, however, is that DPIs can be sensitive to humidity, and *in vitro* studies have shown that the fine particle dose and the actual delivered dose may be negatively affected by storage in humid conditions [14, 15]. Differences in the resistance to humidity between different inhalers have been reported, likely due to the different technical approaches developed to protect the respective dry powder from humidity [15]. A potential decrease of the dose reaching the lungs may eventually impact the clinical outcomes and infer decreased drug efficacy and safety. Still, the full clinical impact has not been indisputably established.

The DPIs available today have differences in shelf-life, time in-use and storage conditions such as protection against humidity (Table 1). In Norway, the drug reimbursement for chronic diseases allows prescription covering a 3 months period, during which patients may store drugs outside the controlled conditions in a pharmacy. However, the actual knowledge of asthma patients’ awareness of and compliance to correct handling of DPIs in daily life is scarce. In the complex approach of achieving full asthma control, this is an important area in need of further investigation. The present patient-reported survey aimed at investigating asthma patients’ storage and retention of dry powder inhalers in everyday use.

**Table 1:** Number of doses, shelf life and storage of the most commonly used multidose dry powder inhalers^a^

Inhaler	Doses	Shelf life (unopened)	Time in-use (opened)	Recommended storage conditions
*Diskus/Accuhaler*	60	1.5–3 years (depending on drug strength)	1.5–3 years	Do not store above 30 °C. Store in a dry place.
				Inhaler is sealed in a foil overwrap, which should only be opened when it is to be used for the first time.
*Easyhaler*	200	3 years	6 months	Store in the original package.
				When in use, do not store above 30 °C and store protected from moisture.
*Ellipta*	30	2 years	6 weeks	Do not store above 25 °C
				Store in the original package in order to protect from moisture.
*Genuair*	60	3 years	90 days	Keep the inhaler protected inside the sealed pouch until the administration period starts.
*Nexthaler*	120	3 years	6 months	Do not store above 25 °C.
				Store in the original package in order to protect from moisture.
*Novolizer*	200	3 years	6 months	Store in the original package.
				When in use, keep the device tightly closed in order to protect from moisture.
*Spiromax*	120	3 years	6 months	Do not store above 25 °C.
				Keep the mouthpiece cover closed after removal of the foil wrapping.
*Turbuhaler*	200	2 years	2 years	Do not store above 30 °C.

^a^ For further details reference to respective inhaler’s Summaries of Product Characteristics (SPCs)

## Methods

### Study design and patient population

A patient reported survey was conducted in collaboration with a pharmacy chain (Boots A/S, Norway). No formal sample size calculation was performed. The pharmacy administered the survey via e-mail to all individuals who had indicated an interest in receiving information from the pharmacy on asthma and/or allergy. Based on the assumption that 50 % of the approximately 20,000 patients included in the pharmacy’s asthma and allergy customer register were asthmatics using DPIs, and with an expected response rate of 10 %, the sample size was estimated to 1,000 patients. Patients had to 1) confirm their diagnosis of asthma; and 2) accept participation in the study to have on line access to the self reported questionnaire. In addition, a patient-confirmed prescription of inhaled treatment by a multi dose DPI during at least 3 months prior to inclusion was required. The survey included questions on basic patient characteristics (such as age, gender, and number of concomitant inhalers for maintenance and as-needed use), and covered also different aspects of inhaler storage (e.g. actual storage conditions and instructions provided prior use) and retention (e.g. usage pattern and expiry date awareness). Primary maintenance defined as the most commonly used inhaler.

### Study objectives

This patient-reported survey aimed at investigating the real life handling of multi dose dry powder inhalers for the treatment of asthma, including storage under non-optimal conditions, use after expiry date, use of concomitant inhalers, and how instructions regarding device handling were given.

### Study variables

The study variables included information such as time on DPI treatment, number of concurrent multi-dose DPI inhalers, frequency of inhaler expiry date checks, use of inhaler after expiry date, storage of inhaler in warm or humid conditions, and information on how patients were instructed in how to store and retain their inhaler devices. Data are presented using descriptive statistics.

### Definition of storage conditions

The following storage conditions, predefined according to study protocol, were considered as risk locations for DPI storage: warm and humid conditions (summer outdoors, bathroom, gym, or indoor swimming pool), humid conditions (winter outdoors), and warm conditions (car, bag).

## Results

A total of 738 patients (median age 41 years; 83 % women) fulfilled the criteria and completed the self-reported questionnaire. Twenty-five percent (25 %) of the patients were between 36–45 years, 30 % were younger and 45 % of the respondents were older than 45 years. A total of 10 % were >65 years. The majority of patients (71 %) had used a DPI for more than 5 years, whereas only 4 % had used their DPI for between 3 months and 1 year. Overall, 81 % reported on having an inhaler for maintenance use. No patient characteristics or other information was available for the non-responders.

The primary maintenance inhaler was stored in the bathroom by 42 % of the patients and either in handbag or pocket by an additional 21 %. For the secondary maintenance inhaler the figures were almost the opposite, with 43 % storage in handbag or pocket, and 18 % in the bathroom. Overall, storage according to the predefined risk locations was reported by 63 % of the patients for the primary, 61 % for the second and 41 % for the third maintenance inhaler, respectively.

**Fig. 1:**
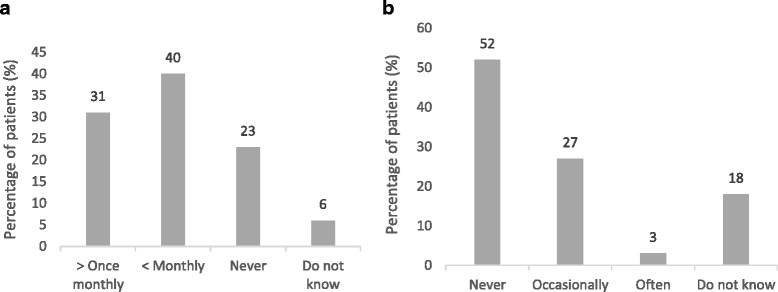
Patient-reported (**a**) frequency of control of DPI expiry date; and (**b**) use after DPI expiry date (per cent of patients)

More than half of the patients (63 %) either did not check the inhaler’s expiry date at all or checked it less frequently than once a month (Fig. 1a). Occasional or frequent use after expiry date was reported by 30 % of the patients, while an additional 18 % were unaware of the expiry date (Fig. 1b). Seventy-nine percent (79 %) of the patients indicated that they used their inhaler until it was empty. There was a trend (non-significant) towards a less thorough check of expiry dates with increased number of inhalers. Age did not impact the level of expiry date checks.

Instructions on how to store and retain their DPI had only been received by 31 % of the patients. Out of these, 45 % had received the information from the package insert, 29 % was informed at the pharmacy, and another 24 % of the patients stated that they had received the information from their doctor and/or nurse. Males and patients younger than 45 years were more likely to have received instructions on DPI storage by the doctor and/or nurse (28 and 26 %, respectively), than females and patients >45 years (23 and 22 %, respectively) (Table 2).

**Table 2:** Patient-reported source of information on dry powder inhaler storage and retention, divided by gender and age below or equal to, or above 45 years

	Male	Female	≤45 years	>45 years
Doctor/nurse, %	28	23	26	22
Pharmacy, %	26	30	29	29
Leaflet, %	43	45	44	46
Other, %	3	2	1	3

Thirty-eight per cent (38 %) of the responding patients stated that they had more than one maintenance inhaler in use at the same time. The majority of these (30 % of the study population) had 2 simultaneous maintenance inhalers, very few (2 %) used four or more inhalers concurrently. The majority of the patients (83 %) reported having a separate reliever medication for symptom control (as needed use), and one third of the patients (29 %) had more than one as needed inhaler in use at the same time. Most common storage place was in a bag, 42 %. Only 4 % of the patients reported storage of their as needed inhaler according to the instructions.

## Discussion

Available DPIs have differences in shelf life, time in-use and storage conditions. Failure to comply with the requirements may impact the functionality of the device. In this patient reported survey on asthma patients treated with multi dose dry powder inhalers, a substantial proportion of asthma patients have their DPIs stored in conditions with risk of exposure to humidity. The patients commonly use their DPIs after expiry date. Further, very few patients had received any information regarding inhaler storage and retention, and for the ones who had, the main source of information was the package insert, not their health care contact or pharmacist. Males were more likely to receive information at their health care center compared to females, which may reflect a gender-linked behavior in health care.

The present study comes with a number of limitations. The design is based on patient reported data only, hence asthma diagnosis and actual treatment with DPI could not be verified, and it is well known that self-reported medication use may be different than actual use. In internet-based research surveys, response representativeness is more important than response rate [16]. In our study, the included population consists of individuals who had actively indicated interest in asthma and/or allergy and may represent an asthma population with more than average knowledge and motivation for asthma treatment. Hence, one could speculate that the knowledge of correct use and storage of inhalers in the general asthma population might be even lower than what is seen in our study. The questionnaire and the storage conditions were defined for the present study only, and were thus not validated or standardized. The gender distribution among the responders can be considered skewed. Although, the study has a low response rate, the results are well in accordance with other studies on real life challenges in asthma care.

Most inhalers have recommendations on storage temperatures and humidity, and some inhalers even need humidity protection storage until start of use (Table 1). Of the currently available DPIs, Diskus and Turbuhaler have the by far longest time in-use, but they may differ in tolerability to humidity. However, this study was not designed to evaluate quality differences between inhalers, but to investigate which conditions an inhaler is exposed to in real life and thus should be able to tolerate. The present study shows that 63 % of the responders store their maintenance inhaler(s) in sub-optimal storage conditions. This is well in keeping with a previous study, where required storage instructions for inhalation capsules was lacking by 84 % of the patients [17].

Concurrent use of multiple inhalers could potentially lead to prolonged use of the inhalers. Fewer inhalations from each inhaler results in a high number of doses left in the inhaler at the expiry date, encouraging continued use. In the present asthma study, a clear majority of the patients reported being more or less unaware of the expiry date of their DPI. This may be further complicated if the patient has more than one type of inhaler with different time in-use.

## Conclusions

The present patient reported survey indicates that two third of the patients store their inhaler devices in suboptimal conditions, and only a minority had received instruction regarding inhaler handling. Two thirds of the study population checked inhaler expiry date less than monthly or not at all. Use of more than one maintenance inhaler simultaneously was common. Overall, this may impact the functionality of the device and result in sub-optimal treatment. Improved communication and patient education to ensure adherence to the different requirements of each inhaler device is needed.
